# Iron intake is positively associated with viral load in antiretroviral naïve Brazilian men living with HIV

**DOI:** 10.1590/0074-02760190350

**Published:** 2020-01-31

**Authors:** Juliana Lauar Gonçalves, Maria Clara Amorim Silva, Eric Henrique Roma, Beatriz Grinsztejn, Alberto dos Santos de Lemos, Nathalia Gorni, Adele Moura Cruz, Cristiane Fonseca de Almeida, Marcel de Souza Borges Quintana, Maria da Gloria Bonecini-Almeida, Patrícia Dias de Brito

**Affiliations:** 1 Fundação Oswaldo Cruz-Fiocruz, Instituto Nacional de Infectologia Evandro Chagas, Serviço de Nutrição, Rio de Janeiro, RJ, Brasil; 2 Fundação Oswaldo Cruz-Fiocruz, Instituto Nacional de Infectologia Evandro Chagas, Laboratório de Imunologia e Imunogenética em Doenças Infecciosas, Rio de Janeiro, RJ, Brasil; 3 Fundação Oswaldo Cruz-Fiocruz, Instituto Nacional de Infectologia Evandro Chagas, Laboratório de Pesquisa Clínica em DST e AIDS, Rio de Janeiro, RJ, Brasil; 4 Fundação Oswaldo Cruz-Fiocruz, Instituto Nacional de Infectologia Evandro Chagas, Centro Hospitalar, Rio de Janeiro, RJ, Brasil; 5 Fundação Oswaldo Cruz-Fiocruz, Instituto Nacional de Infectologia Evandro Chagas, Plataforma de Pesquisa Clínica, Rio de Janeiro, RJ, Brasil

**Keywords:** HIV, iron intake, nutrition, ART naïve

## Abstract

**BACKGROUND:**

Iron homeostasis contribute for the human immunodeficiency virus (HIV) pathogenesis.

**OBJECTIVES:**

We assessed the iron intake pattern in antiretroviral naïve Brazilian men living with HIV correlating with clinical and nutritional parameters.

**METHODS:**

The iron consumption mean was estimated according to a food frequency questionnaire (FFQ), and a 3-day food record (3dFR) submitted to the patients. HIV viral load, CD4^+^ T cell counts, serum iron, haematological and anthropometrics parameters were recorded.

**FINDINGS:**

Fifty-one HIV-infected adult men naïve for antiretroviral therapy (ART) were enrolled. The mean age of participants was 35 (SEM ± 1.28) years old, with mean time of HIV-1 infection of 1.78 (0-16.36, min-max) years. Majority (41.18%) had complete secondary, and 21.57% had tertiary educational level. The income was around 1x (54.90%) to 2x (41.18%) minimum wage. Fifty-four percent showed normal weight, while 40% were overweight. The patients showed normal mean values of haematological parameters, and mean serum iron was 14.40 µM (SEM ± 0.83). The FFQ showed moderate correlation with the 3dFR (ρ = 0.5436, p = 0.0009), and the mean values of iron intake were 10.55(± 0.92) mg/day, recorded by FFQ, and 15.75(± 1.51) mg/day, recorded by 3dFR. The iron intake, recorded by FFQ, negatively correlated with serum iron (ρ = -0.3448, p = 0.0132), and did not have influence in the CD4^+^ T cell counts [e.B 0.99 (0.97-1.01, 95% confidence interval (CI), p = 0.2]. However, the iron intake showed a positive effect in HIV viral load [e.B 1.12 (1.02-1.25, 95%CI), p < 0.01].

**MAIN CONCLUSIONS:**

This study draws attention for the importance of iron intake nutritional counseling in people living with HIV. However, more studies are required to clarify the association between high iron intake and HIV infection and outcome.

Host iron status and infectious diseases relationship has been established in animal models with evidence of potential associations demonstrated in humans.^1^ Iron is an essential trace element present in biological systems, and is vital for pathogens as well as host survival.^2^ In human immunodeficiency virus (HIV)-1 infection, iron metabolism is altered through a complex mechanism in all stages of the disease. Iron accumulation can be detected in all stages of the disease in several tissues.^3^ Ferritin concentration also increases with HIV disease progression and a decreased concentration of haemoglobin and serum iron has been extensively documented.^3^ Since the beginning of the HIV-1 pandemic, anaemia has been described as a common haematological abnormality associated with poor prognosis.^4^

Concerns about nutrition in individuals with HIV infection often leads to the high consumption of minerals and trace elements by infected individuals, besides the increased intake of iron rich foods. However, some reports have shown that increased iron bioavailability might be related to progression to acquired immunodeficiency syndrome (AIDS).^5^ Furthermore, iron overload might have a negative impact on CD4^+^ T cell counts.^6^ Among the mechanisms responsible for the poor prognosis associated with iron overload is the oxidative stress which may contribute for CD4^+^ T cells depletion and low antioxidants storage,^7^ as well as the risk of opportunistic infections such as tuberculosis, which are enhanced in this scenario. *In vitro* studies showed HIV-1 replication impairment in iron depleted environments.^8^ In addition, dysregulation of hepcidin, a key hormone controlling iron metabolism, causes accumulation of this trace element inside the cells, leading to availability of free iron for viral replication.^9^

The recommendation for iron supplementation in a non-iron-deficient anaemias in HIV-1 infected patients might be considered a double-edge sword since the excess of iron consumption might increase the viral replication, while serum iron deficiency leads to increased mortality rates. However, the evaluation of the role of iron intake in people living with HIV/AIDS is difficult. First, because anaemia is very common in HIV infection (20-80%).^10^ Second, HIV-infected patients start the antiretroviral therapy (ART) as soon as the infection is diagnosed, which could mask the negative effects of iron in viral load and CD4^+^ T cell counts. In this scenario, studies addressing the role of iron in HIV infection before ART implementation could elucidate the effects of this trace mineral in disease outcomes. Many reports draw attention to the effects of iron supplementation in HIV-1 infected individuals.^11^ ^,^ ^12^ ^,^ ^13^ ^,^ ^14^ However, in light of our knowledge, none of these reports correlated the habitual iron ingestion with disease prognosis.

In the present study, we evaluated iron intake in antiretroviral naïve men living with HIV, correlating iron consumption with viral load and CD4^+^ T cell counts.

## SUBJECTS AND METHODS

*Subjects and selection criteria* - We conducted a cross-sectional observational study with patient inclusion from March to November 2010. Adult men (over 18 years) with asymptomatic HIV-infection, treatment-naïve for ART were recruited at the Instituto Nacional de Infectologia Evandro Chagas (INI), a reference centre for treatment of infectious diseases at Fiocruz, Rio de Janeiro, Brazil. The subjects included in this study are regularly followed at INI/Fiocruz and participate in the HIV Study Cohort implemented in the Institute since 1986. Cohort procedures and results have been published elsewhere.^15^ Participation included male gender in follow-up at INI/Fiocruz, with a positive serology, age of 18 to 60 years, and ART treatment naïve. Women were not included in the study because the interference of female hormones and endometrial bleeding in iron homeostasis. Exclusion criteria included use of female hormones, underweight, diarrhea at the time of data collection any physical impairment impeding assessment of anthropometric measurements, and presence of co-morbidities that could change the metabolism, such as cancer, hepatic cirrhosis, viral hepatitis, pulmonary or kidney disease, and tuberculosis. Patients with underweight were excluded because direct relationship between nutritional status and immune response, that could interfere with CD4+ T cell counts and viral load in these individuals.

*Study data collection* - The patients were seen for a nutritional visit where clinical, anthropometrics and demographic data were collected. In parallel, the patients answered a semi-quantitative food frequency questionnaire (FFQ) and the 3-day food record (3dFR) for iron intake estimation, and blood was collected by venipuncture.

For the demographic data, educational level was classified using the International Standard Classification of Education (ISCED),^16^ according to the categories: 0- Early childhood education; 1-primary education; 2-lower secondary education; 3-upper secondary education; 4-post-secondary non-tertiary education; 5-short-cycle tertiary education; 6-bachelor’s or equivalent level and 7-master’s or equivalent level (ISCED 2011). Household income was represented in minimum wages, where minimum wage corresponded to US$ 294.80 in 2010 (Brazilian Central Bank).^17^

For anthropometrics data, body weight and height were measured to calculate body mass index (BMI = body weight/height^2^), and nutritional status was classified according to criteria of the World Health Organization.^18^

CD4^+^ T cell counts and viral load were obtained from the medical records three months before or after inclusion in the study. Therefore, for these analyses, a convenience sample was used and some missing values reduced the sample size in these parameters. Patients missing CD4^+^ T cell counts were excluded from the study. Total iron binding capacity, and serum iron measurements were performed at the Laboratory of Clinical Pathology at INI/Fiocruz. Transferrin values were obtained from the total iron biding capacity (TIBC) using the formula: Transferrin = (0.8 x TIBC) - 43. The patients’ blood count values were obtained from the medical records in a maximum interval of 60 days from the nutritional visit. Red blood cell (RBC) counts, haemoglobin (Hb), hematocrit (HCT), mean corpuscular volume (MCV), mean corpuscular haemoglobin (MCH) and mean corpuscular haemoglobin concentration (MCHC) were measured from blood samples collected into tubes containing EDTA. These measurements were used to define the levels of anaemia: Hb 10.5 -12.99g/dL - mild; Hb 8.0-10.49g/dL - moderate; Hb < 8.0g/dL severe. Serum iron deficiency was defined as < 8.6 μM and serum iron overload > 30 μM. The reference limits to haematological data were: MCV, 80-96 fL; MCH 27-33 pg; MCHC, 32-36 as pointers; low MCV (< 80 fL) was indicative of microcytosis; high MCV (> 96 fL) was indicative of macrocytosis; low MCH (< 27 pg) indicates hypochromia.^19^

For iron intake evaluation, a semi-quantitative, retrospective FFQ was applied, recording the patient dietary intake through the last month prior to the nutritional visit. The FFQ was validated for Brazilian population based on National Study of Family Expenses.^20^ The FFQ was composed by a list of 71 foods. Some foods of usual consumption were added such as soy, textured soy protein, chickpeas, canned fish, okra, zucchini, margarine, olive oil, milk cream, jelly, and coconut water. Iron source foods were considered in FFQ such as meats, meat products, fish, iron enriched processed foods, and rich iron vegetables such as legumes.

Dietary iron values were calculated by sum of consumed food items considering weight and frequency of consumption. Use of dietary supplements containing iron was self-reported. Subjects were asked to write the name of any supplements they were currently using that contained iron, the frequency of consumption and the dose. The dose of supplemental iron (as elemental iron) was confirmed by checking supplement package or the manufacturers’ websites. To estimate supplemental mean iron intake per day, the total amount of iron from all supplements consumed per week was divided by seven days. Supplemental iron consumption was added to dietary calculations to determine the mean of iron intake per day. Iron was expressed in mg/day. For iron intake evaluation using a 3dFR, the enrolled individuals filled a specific questionnaire with instructions to register all consumed food and drinks through three alternate days, where one was in a weekend day, and two in working days. All study patients were instructed on how to fill the forms correctly reporting consumed foods. This method relied on registering foods during the time of consumption, in this way eliminating recall bias.^21^

*Statistical analysis* - The program Statistical Package for Social Sciences (SPSS) for Windows version 16.0 and GraphPad Prism 6.0 for Macintosh were used to store the database and perform statistical analysis. Categorical variables were described in frequencies and numerical variables were expressed as mean and standard error, or median and minimum-maximum. To evaluate association between iron intake from FFQ versus 3dFR, and iron intake versus serum iron, we performed Spearman´s rank correlation between these parameters. For the univariate and multivariate analysis, generalised linear models were applied assuming Gamma Distribution for the continuous variables CD4^+^ T cell counts and viral load applying log link function. The multivariate analysis was calculated considering the time of HIV infection, as represented in the Table I. eB as stated in Table II stands for the effect of iron intake or serum load on absolute CD4 cell counts or viral load, in decimal form. The level of significance was set to p < 0.05.

*Ethics* - All participants provided written informed consent. The project was approved by the Instituto Nacional de Infectologia Evandro Chagas Research Ethics Committee under protocol number (CAAE) 0068.0.009.000-09 and all methods were carried out in accordance with the local guidelines and regulations. This study was conducted according to the Declaration of Helsinki. Patients who had unfavorable nutritional profiles or inadequate food intake were invited to have educational activity with nutritional guidance.

## RESULTS

*Patient characteristics* - In this report, we analysed the influence of iron intake in antiretroviral naïve Brazilian men living with HIV, in an observational cross-sectional study. Sixty-nine patients were eligible for the study and recruited. Due to critical missing data, treatment interference or nutritional status, 18 eligible subjects (28% of cases) were excluded. Among excluded patients, one started ART before blood collection, six had missing blood collection data, three did not fulfill the FFQ, seven had missing analysis of serum iron, and one had missing data for CD4^+^ T cells. The data representing demographics, anthropometrics, haematological, and iron intake are in Table I. The mean age of the participants was 35 ± 1.28 years old. The income varied between 1x (n = 28; 54.90%), 2x (n = 21; 41.18%), or 4x (n = 2; 3.92%) minimum wage. Regarding education level, one patient (1.96%) had lower secondary education, three (5.88%) had master’s degree or equivalent, six (11.76%) had short-cycle tertiary, nine (17.65%) had upper secondary education, 11 (21.57%) had bachelor’s or equivalent degree, and 21 (41.18%) had post-secondary non-tertiary education level. The mean time of HIV infection was 1.78 years. According to BMI, 27 (54%) patients had normal weight, 20 (40%) were overweight, and three (6%) showed obesity.

**TABLE I:** Characteristics of antiretroviral naïve Brazilian men living with human immunodeficiency virus (HIV)-1

Variables	Findings
Demographics	
Age (years), n = 51^*a*^	35 (± 1.28)
Income (minimum wage), n = 51^*b*^	
1x	28 (54.90%)
2x	21 (41.18%)
3x	0 (0%)
4x	2 (3.92%)
Education level (categories), n = 51^*b,c*^	
0-early childhood	0 (0%)
1-primary	0 (0%)
2-lower secondary	1 (1.96%)
3-upper secondary	9 (17.65%)
4-post-secondary non-tertiary	21 (41.18%)
5-short-cycle tertiary	6 (11.76%)
6-bachelor’s or equivalent	11 (21.57%)
7-master’s or equivalent	3 (5.88%)
Diagnosis of HIV-1 infection (years), n = 51^*d*^	1.78 (0.00-16.36)
Anthropometrics parameters, n = 50^*b*^	
BMI (kg/m^2^)	
Normal weight	27 (54%)
Overweight	20 (40%)
Obesity	
Haematological and iron parameters, n = 45^*a,d*^	
RBC (1 x 10^6^ cells/mm3)	4.90 (3.71-6.89)
MCV (μm3)	86.30 (69.7-98.4)
MCH (pg)	29.2 (21.0-34.3)
MCHC (g/dL)	33.8 (30.2-35.9)
Haemoglobin (g/dL)	14.49 (± 0.17)
Hematocrit (%)	43.08 (± 0.49)
Transferrin (mg/dL)	209.0 (108.2-350.6)
Serum iron (µM), n = 51^*a*^	14.40 (± 0.83)
Iron intake (mg/day)^*a*^	
FFQ, n = 51	10.55 (± 0.92)
3dFR, n = 34	15.75 (± 1.51)

*a*: mean and ± std. error; *b*: count and percentage; *c*: international standard classification of education 2011; *d*: median and range (minimum - maximum). BMI: body mass index; RBC: red blood cells; MCV: mean corpuscular volume; MHC: mean corpuscular haemoglobin; MCHC: mean corpuscular hemoglobin concentration.

*Patient haematological parameters, serum iron levels, and iron intake* - The mean of RBC counts was 4.90 (3.71-6.89) x 10^6^ cells/mm^3^, while MCV, MCH, and MCHC were 86.30 (69.7-98.4) µm^3^, 29.2 (21.0-34.3) pg, 33.8 (30.2-35.9) g/dL, respectively. The values of haemoglobin, hematocrit and transferrin were 14.49 ± 0.17 g/dL, 43.08 ± 0.49 %, and 209.0 (108.2-350.6) mg/dL, respectively. The majority of patients (n = 47; 92%) did not fulfill the criteria for anaemia. Of the four anaemic patients, one presented moderate, and three mild anaemia. The mean serum iron was 14.40±0.83 µM, and the iron intake recorded by FFQ was 10.55 ± 0.92 mg/day, while iron intake recorded by 3dFR was 15.75 ± 1.51 mg/day. Three patients were serum iron deficient, and another one presented iron overload. The iron intake analysis of patients with anaemia, showed that only one was in use of iron supplementation.

**Fig. 1:**
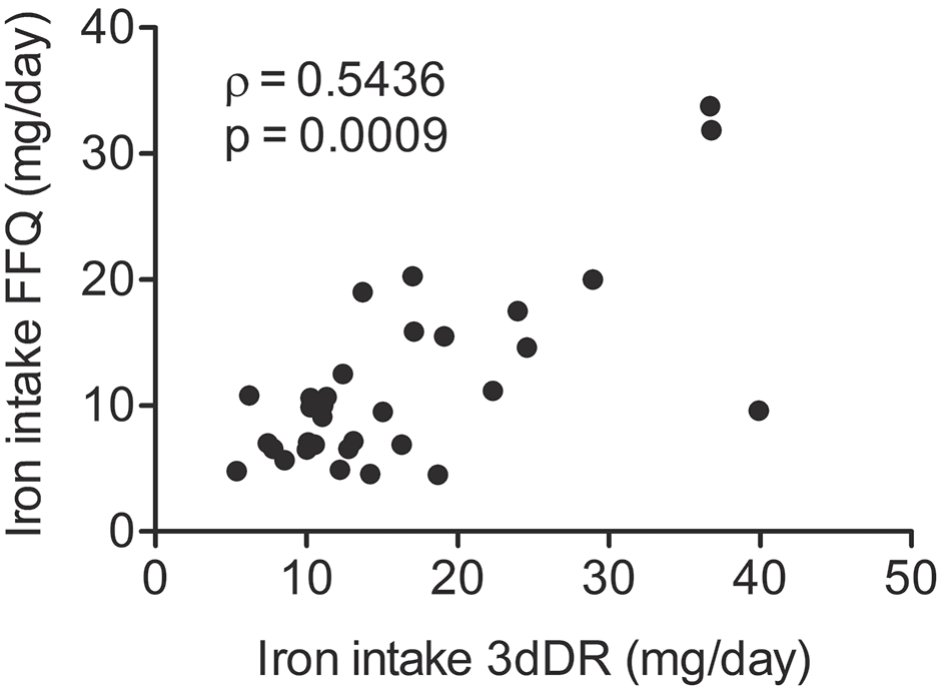
correlation between iron intake data collected from food frequency questionnaire (FFQ) versus 3-day food record (3dFR) method. Spearman’s rank correlation between FFQ vs 3dFR, ρ = 0.5436, p = 0.0009. The data were expressed as the mean iron intake in milligrams per day (mg/day).

*Iron consumption and correlation with disease status* - As this study analysed convenience samples, we used data from FFQ to estimate the mean iron consumption in a higher number of patients (n = 51). As 3dFR is a more robust method to quantify food consumption, we compared the iron consumption data from FFQ versus 3dFR, since we had lower number of patients that returned the 3dFR (n = 34). Spearman coefficient analysis showed moderate correlation between both methods with high statistical significance (ρ = 0.5436, p = 0.0009) (Fig. 1). This result allowed us to use the FFQ in further analyses to increase the statistical power of the study, given the higher number of patients evaluated by this method.

**Fig. 2:**
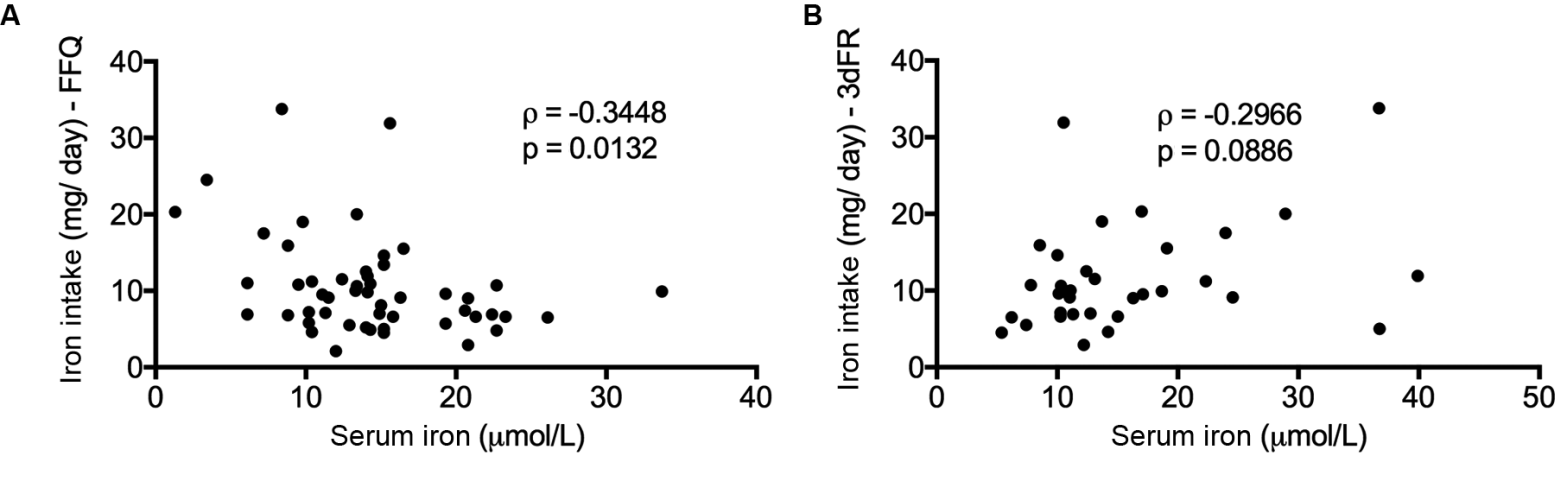
correlation of iron intake with serum iron. (A and B) Spearman’s rank correlation between iron intake by food frequency questionnaire (FFQ) and 3-day food record (3dFR), and serum iron (pooled CD4 cell count groups), ρ = -0.3448, p = 0.0132, and ρ = -0.2966, p = 0.0886, respectively. The data were expressed as the mean of the iron intake in milligrams per day (mg/day), and serum iron was expressed as μM.

Negative correlation was observed between iron intake and serum iron by FFQ (ρ = -0.3448, p = 0.0132) (Fig. 2A), while no correlation between serum iron and iron intake collected in the 3dFR was observed (ρ = -0.2966, p = 0.0886) (Fig. 2B).

We further performed analyses to investigate the effects of the iron intake and serum iron in CD4^+^ T cell counts and viral load (Table II). We observed a positive effect of iron intake on viral load, in both FFQ and 3dFR methods in uni or multivariate analyses, where the time of HIV infection was considered as confounding factor. The univariate analysis of FFQ showed an eB 1.15 (1.04-1.31, CI95%), p < 0.01, and 3dFR eB 1.1 (1.02-1.2, CI95%), p = 0.01, indicating an increment of 15% and 10% in viral load per milligram of iron intake, calculated in FFQ and 3dFR methods, respectively. The adjusted analysis considering the time of HIV infection showed similar results, eB 1.12 (1.02-1.25, CI95%), p < 0.01, in FFQ, and eB 1.09 (1-1.2, CI95%), p = 0.02, in 3dFR method. These results indicated no interference of the time of HIV infection in the effect of iron intake in the viral load. We evaluated the effect of the serum iron in CD4^+^ T cell counts and viral load, in association with iron intake. We observed an association of serum iron with CD4^+^ T cell counts and viral load in the patients (eB 1.01 (1-1.01, CI95%), p < 0.01, and eB 0.98 (0.97-1, CI95%), p = 0.01, respectively) in both uni- and multivariate analyses, showing an association between circulating iron and disease status (Table II). The analysis considering the time of HIV infection did not change this effect for both CD4^+^ T cell counts and viral load parameters. On the other hand, we observed a positive association of serum iron with CD4 T cell counts in both uni- and multivariate analyses (eB 1.01 (1-1.01, CI95%), p < 0.01), and a negative association with viral load also in both analyses (eB 0.98 (0.97-1, CI95%), p = 0.01, which means a decrease of 2% in viral load for each additional 1 µM of serum iron.

**TABLE II:** Iron parameters, CD4 cells and viral load correlations of antiretroviral naïve Brazilian men living with human immunodeficiency virus (HIV)

Variables	CD4 cells				Viral load (HIV RNA copy/mL)			
	Univariate		Multivariate		Univariate		Multivariate	
	eB (CI-95%)	p value	eB (CI-95%)	p value	eB (CI-95%)	p value	eB (CI-95%)	p value
Iron intake (FFQ) (n = 51)	0.98 (0.96-1)	0.11	0.99 (0.97-1.01)	0.2	1.15 (1.04-1.31)	< 0.01	1.12 (1.02-1.25)	< 0.01
Iron intake (3dFR) (n = 34)	0.99 (0.97-1.01)	0.22	0.99 (0.97-1.01)	0.29	1.1 (1.02-1.2)	0.01	1.09 (1-1.2)	0.02
Serum iron (n = 51)	1.01 (1-1.01)	< 0.01	1.01 (1-1.01)	<0.01	0.98 (0.97-1)	0.01	0.98 (0.97-1)	0.01

CI-95%: confidence interval 95%; eB: effect of the variables in the CD4 cells numbers or in viral load; FFQ: food frequency questionnaire; 3dFR: 3-day food record. The multivariate analysis was performed to adjust the analysis considering the time of HIV infection.

## DISCUSSION

To our knowledge, this is the first study evaluating regular dietary iron intake in HIV-1 infected ART-naïve individuals which explores potential associations with disease status. We showed a positive association between iron intake and viral load. For each arbitrary unit of iron consumed, there is a median increase of 12% in the serum viral load of patients, considering confounding factors, such as the duration of HIV infection. However, no association between iron intake and CD4^+^ T cell counts was observed. Interestingly, the iron intake did not seem to be positive correlated with serum iron levels. In contrast, we observed a negative correlation between iron intake and serum iron.

Individuals with similar income and education level composed the population of this study. As both groups presented similar characteristics among these parameters, these patients did not present financial and education limitations to acquire food sources and information. Moreover, these individuals are followed in regular outpatient care, in the HIV Study Cohort from INI/Fiocruz, presenting easy access to high quality healthcare. In fact, through the Brazilian Ministry of Health, HIV infected patients in Brazil have free access to treatment and medications to control the disease. In this scenario, it is unlikely that other factors would affect the results of the present study, including limited access to health services or food insecurity.

Iron metabolism and HIV infection have been extensively studied in the literature, and a number of reports have investigated potential interactions as reviewed.^22^ The majority of these studies have pointed for increased morbidity in iron deficient populations, particularly infants and young children.^23^ However, few studies evaluated iron ingestion in non-deficient conditions, especially considering individual diets.

In this work, we used the FFQ method to estimate the daily iron intake. Since 3dFR is a more reliable to quantify food nutrients, we compared the results generated by FFQ versus 3dFR to increase data reliability. However, we could not use only 3dFR data because the reduced number of patients submitted to this method (n = 34) compared to patients submitted to FFQ (n = 51). Indeed, many studies showed good correlation between FFQ and 3dFR. Perreault et al.^24^ showed that FFQ is a reliable method to assess bone nutrients in pregnant women. Kent et al.^25^ validated a FFQ to estimate flavonoid intake comparing to a 4-day food record with good Spearman correlation between methods. In another study, FFQ was shown to be a good dietary assessment tool to estimate the intake of 103 food items, including iron analysis.^26^ Corroborating these data, we showed a good correlation between FFQ and 3dFR regarding iron intake in our model, suggesting that FFQ could be a good method to estimate the consumption of this trace element in our study.

*In vitro* studies showed that increased levels of iron promotes cellular death in T-lymphoid cell line infected with HIV as well as viral replication.^6^ The iron chelation restores cell viability and decreases viral replication, showing essential role of iron in the infection. In contrast to our work, Banjoko et al.^7^ showed a negative correlation of serum iron and CD4^+^ T cells in African population. However, the authors did not correlate these data with viral load. In addition the cohort of this study was composed by 60% of women, and the mean serum iron of the study subjects was significantly higher than found in our work. These differences, added to distinct population profiles could interfere in CD4 and serum iron counts, could explain contradictory results.

Some reports have shown the effects of iron supplementation in people living with HIV-1. James et al.^11^ showed no effect of iron supplementation in African malnourished HIV-infected patients. Semba et al.^12^ compared the effect of a daily micronutrient supplement containing iron in female drug users for 12 months. Among one-third of HIV-positive individuals were on ART at baseline, and any event related to mortality, disease progression or CD4 cell counts and viral load changes were reported. The iron supplementation did not change the HIV RNA levels in women drug users, despite reducing anaemia and ameliorating iron status.^13^ Olsen et al.^14^ reported no adverse effects on HIV-1 viral load in adults after the ingestion of 60 mg of iron given twice weekly over four months. However, effects due the use of higher doses of iron cannot be excluded. Differently of these studies, our work focused in determine the effect of habitual iron intake in nourished men, to exclude possible interferences regard sexual hormones and nutritional status in the iron metabolism and immune response. Therefore, we could analyse the role of iron intake in HIV infection parameters with less interference of possible confounding factors.

In our observational study, the evaluation of iron ingestion, through a food frequency questionnaire, might represent the food habit of the individuals. Therefore, this method can estimate the iron intake in an extended period, and despite to be a cross sectional study, we could observe an adverse effect of iron accumulation over the time. Confirming the reliability of this method, the results observed in 3dFR method were similar to those of FFQ.

There is no consensus on specific micronutrients recommendations for people living with HIV (PLHIV), although many authors argue that, since HIV infection is a chronic inflammatory condition, some differences from general population guidelines may apply. Hence, PLHIV are encouraged to follow the same principles of healthy eating as recommended for everyone. To ensure an optimal intake of micronutrients that could be not well absorbed, such as iron, selenium and vitamin B_12_, PLHIV are advised to include a variety of fruits, vegetables, whole grains, low-fat dairy and lean protein foods in diet.^27^ However, they tend to ingest vitamins and minerals supplements without medical prescription, believing that this practice might improve their quality of life. Studies supporting the nutritional guidelines of micronutrients for this population are important. Nutritional repletion of micronutrients has been recommended, albeit, deficiencies as well as excess of nutrients, adversely might affect immune response. According to the American Dietetic Association for nutrition intervention and HIV infection (2010),^27^ anaemias should be evaluated prior to nutrition intervention, such as dietary iron and supplementation of folate or vitamin B_12_, and dietary adequacy of vitamins and minerals and its potential toxicity and interactions with anti-retrovirals should be considered before recommending supplementation.

Quiros-Roldan et al.^28^ showed that ART might change the iron metabolism in PLHIV, increasing the concentration of serum iron and percentage of transferrin saturation. This is an important point of our work, since we analysed patients naïve for ART, eliminating any possible confounder effect of the anti-retroviral therapy in iron metabolism.

In our study, the frequency of anaemia was low (8% of patients) and only one patient presented iron deficiency. Thus, iron-deficiency anaemia was minimised as a confounder for the possible correlation of a deleterious role of iron deficiency in people living with HIV. Interestingly, we observed a negative correlation between iron intake and serum iron. The regulation of iron concentration in the body depends of a balance through the intestinal absorption, since this trace mineral is not regularly secreted. The hormone hepcidin seems to be the link between nutritional, inflammatory and infectious regulation of iron status. This peptide hormone, synthesised and secreted by the liver in response to high serum iron, downregulates ferroportin at the protein level and thereby limits iron absorption from the diet. Hepcidin regulation may explain why iron consumed in the diet does not reflect serum iron, especially in the context of infectious diseases. High hepcidin levels may contribute to the establishment and maintenance of viral set-point, which is a predictor of aids progression and death.^29^ Besides controlled by hormones, iron bioavailability might be regulated by combined foods during meal. For example, ascorbic acid increases, while calcium decrease the iron absorption. These factors might interfere with serum iron concentration, and possibly explain an inverse correlation between iron intake and serum iron observed in our study.

In our work we showed a positive association between the iron intake and viral load, and this trace element has been reported as essential for HIV replication.^30^ At least, five steps of HIV-1 replication require iron: reverse transcribed RNA into DNA requires dNTPs that are generated by the iron-dependent protein ribonucleotide reductase; transcription of viral genes are mediated by nuclear factor kappa B (NF-κB) that can be activated indirectly by iron in a mechanism where iron generates reactive oxygen species (ROS) activating inhibitor of kappa B (IκB), and consequently NF-κB; low cellular iron inhibited HIV-1 transcription; inhibiting the amino acid hypusine (HY), blocks HIV-1 replication, and this amino acid synthesis is iron dependent; finally, the translation of capsid proteins into mature virions requires the host cell protein ABCE1, an iron-binding ATPase. In fact, our results showed a positive correlation between iron intake versus viral load, indicating a possible increase of iron bioavailability for viral replication.

Our results did not point for a role of iron intake in CD4^+^ T cell counts. However, Banjoko et al. showed depletion of CD4^+^ cells due to an unbalanced iron metabolism and oxidative stress in HIV infected individuals, culminating in a poor disease prognosis.^7^ Possibly, others factors not analysed here might interfere with CD4^+^ T cell counts, including the genetics of each individual and HIV subtypes.

The present study showed some limitations, and the results must be carefully analysed. The limited number of patients could generate a bias in the analysis. In addition, despite FFQ showing good correlation with 3dFR in our model, 3dFR is still the gold standard method to quantify food nutrients. Because the FFQ format, we were unable to calculate the iron bioavailability, that would allow to us quantify the iron intake by absorption. Indeed, some food combination might increase or decrease iron absorption. As we used convenience samples, we were not able to evaluate other components of iron metabolism such as hepcidin and ferropotin. Future studies including higher number of participants, and including analysis of hepcidin and ferroportin might clarify the effects of iron ingested by people living with HIV and correlation with disease status and progression. Based on results of this work, we do not recommend iron supplementation for HIV-infected individuals with normal levels of iron and non-anaemic. In our perspective, the iron dietary recommendation for men infected with HIV should range between estimated average requirement and recommended dietary allowance values according to age. However, because the low number of patients in different iron status conditions, among other factors such was iron absorption and hormones, this study should be carefully analysed. The study supports the need for reevaluation of iron ingestion guidelines in PLHIV who are non-iron deficient.
